# The effect of IVF pregnancies on mortality and morbidity in tertiary unit

**DOI:** 10.1186/1824-7288-39-17

**Published:** 2013-03-12

**Authors:** Gülcan Turker, Emek Doger, Ayşe Engin Arısoy, Ayla Günlemez, Ayşe Sevim Gökalp

**Affiliations:** 1 Department of Pediatrics, Neonatology Division, Faculty of Medicine, Kocaeli University, Umuttepe Campus, Kocaeli 41380, Turkey; 2 Department of Obstetric and Gynecology, Faculty of Medicine, Kocaeli University, Umuttepe Campus, Kocaeli, 41380, Turkey

## Abstract

**Background:**

There are several studies that have shown an increased risk of premature birth and developmental abnormalities with in vitro fertilization (IVF); however, the data on preterm mortality and morbidity are limited.

**Aim:**

Our aim is to investigate whether IVF had an effect on the mortality and morbidity in neonates admitted to the neonatal intensive care unit.

**Methods:**

A total of 940 term and preterm babies who were admitted to the intensive care unit over a period of 2 years were enrolled. Of these, 121 babies were born after IVF and 810 were born after a natural conception and 9 were born after ovulation induction. Of these, 112 preterm babies were born after IVF and 405 preterm babies were born after a natural conception.

**Results:**

In the IVF group, the gestational age and birth weight were significantly lower than in the non-IVF group. Additionally, in the IVF group, multiple births were significantly higher than in the non-IVF group. IVF pregnancies increase preterm delivery but did not increase preterm mortality, and preterm morbidity did not differ among groups, except for intraventricular hemorrhage (IVH). Gestational age was shown to be the primary risk factor for IVH using a logistic regression analysis. Also when newborns at gestational age <32 weeks were compared using regression analysis, gestational age was the major risk factor for IVH.

**Conclusion:**

IVF appears to be associated with premature delivery and the known risks associated with prematurity.

## Introduction

Assisted reproductive technique rates are increasing worldwide. Parallel to the rise in assisted reproductive technique rates, publications that demonstrate intrauterine growth retardation, premature birth, perinatal mortality, very low birth weight, and congenital anomalies as contributing factors for poor perinatal outcomes have also increased [1-7]. The mortality of premature infants has decreased in our country and in neonatal intensive care units (preterm mortality 5%) over the past decades; yet, our country still has higher mortality than other developed countries [8,9]. In particular, multiple gestations are increased after in vitro fertilization (IVF) [10-12]. The risk for low-birth-weight and very-low-birth-weight infants or adverse perinatal outcomes is increased in singletons and multiples after IVF when compared to natural conception [13-15].

Studies that have investigated the effects of IVF on neonatal mortality and morbidity are increasing recently. However, studies on the outcome of preterm infants, especially very-low-birth-weight infants, after IVF are limited. In this study, we aimed to study whether IVF had an effect on the mortality and morbidity in neonates admitted to the neonatal intensive care unit.

## Methods

### Study population

The University Hospital is the only referral center in this region with a 20-bed critical care neonatal unit and an IVF center. Thus, many high-risk pregnancies (including fetuses with cardiac defects) are referred to this obstetric department, and nearly 50% of these newborns are hospitalized. All of the newborns treated in the neonatal intensive care unit between 2009 and 2010 were included in this study. There were 121 infants born after IVF pregnancies including intracytoplasmatic sperm injection, 9 infants born after ovulation induction and 810 infants born after naturally conceived pregnancies. After the 9 infants with ovulation induction were excluded from our analysis and 931 newborns were finally studied.

### Data Collection

All medical records were retrospectively collected through specially designed. These data were entered into a computerized relational database. For each newborn, the basic demographic information, biological data, and clinical outcomes (diagnosed or suspected) during the observation period were recorded. The local ethical committee gave approval to the study.

Preterm infants were defined as a gestational age below 37 weeks.

Small for gestational age was defined as a birth weight below the 10th percentile.

Patent ductus arteriosus (PDA): The occurrence of hemodynamic PDA was defined according to echocardiographic criteria (left atrial to aortic root ratio >1.5, pulsed Doppler signal within the duct and ductal diameter >1.5 mm in the first 30 hours after delivery, pulsatile transductal flow (Vmax) <1.8 m/sec, reverse end-diastolic flow in the descending aorta/mesenteric artery) and/or clinical signs and symptoms of significant left-to-right ductal shunting, such as an active precordium, bounding pulses, wide pulse pressure, and systemic hypotension. According to our neonatal intensive care unit standards, preterm infants at gestational age <32 weeks had screening echocardiograms for PDA between days one and four of life routinely. Indomethacin or ibuprofen infusion was given to preterm newborns if clinical symptoms were present and if the echocardiographic criteria for hemodynamic PDA were met.

Intraventricular hemorrhage (IVH): Cranial sonograms were performed routinely, daily in the first week of life and weekly thereafter until hospital discharge, or more frequently if clinically indicated for all preterm newborns. IVH was classified from grade I to IV according to the grading system of Papile et al.

Bronchopulmonary dysplasia (BPD): The classic diagnosis of BPD was identified as oxygen dependency at 36 weeks post-conception age.

Retinopathy of prematurity (ROP): An ophthalmologic examination was performed in the fourth week of life. It was repeated every two weeks by the same examiners. After pupillary dilatation, indirect ophthalmoscopy was performed. Fundoscopic findings were recorded in accordance with the International Classification of ROP. Examinations were repeated until the retina was completely vascularized or until the retinopathy was stable for at least 3 months.

Necrotizing enterocolitis (NEC): Necrotizing enterocolitis was diagnosed according to Bell’s classification stage >2.

Sepsis: Sepsis was dignosed when positive blood culture and CRP.

Criteria for rescue treatment of respiratory distress syndrome (RDS): Early rescue treatment with a surfactant was given to preterm infants with respiratory distress who required continuous nasal positive pressure or assisted ventilation and did not present with radiological evidence of another disease process.

Life-threatening congenital anomalies are chromosomal abnormalities (trisomy 13, 18), renal agenesis, pulmonary hypoplasia, diaphragm hernia and the other major malformations.

### Statistical analysis

For many variables in the collected data, the standard deviations were wide and the distributions were not normal. Therefore, a statistical analysis was performed using medians and ranges. Differences between groups were analyzed using the Mann–Whitney U test or the chi-squared test. Risk factors of mortality were evaluated with binary logistic regression analysis in all preterms. Independent risk factors of mortality in all of the newborns were IVF, gestational age, SGA, preterm premature rupture of membranes (pPROM), preeclampsia, Apgar score at 5 minutes, duration of surfactant administration, congenital anomalies (non-life-threatening or life-threatening) and multiple gestations. The risk factors for morbidity, including the administration of surfactant, need of mechanic ventilation, PDA and IVH, were evaluated with a binary logistic regression analysis only in preterm newborns. The independent risk factors of surfactant, the need of mechanic ventilation, PDA and IVH were IVF, gestational age, SGA, preterm premature rupture of fetal membranes (pPROM), preeclampsia, Apgar score at 5 minutes, duration of surfactant administration and multiple gestations. Additionally, the risk factors of IVH in newborns at gestational age <32 weeks were evaluated using a binary logistic regression analysis. Independent risk factors of IVH in newborns at gestational age <32 weeks were IVF, gestational age, multiple gestations and antenatal steroid administration. The variables were studied as independent risk factors in logistic regression analyses and were significantly different in the IVF group when compared to the non-IVF group.

Statistical analyses were performed using the Statistical Package for Social Sciences for Windows (SPSS, Inc., Chicago, Illinois, USA). Statistical significance was defined as a p-value < 0.05.

## Results

The distributions of the study population according to the gestational age at birth and the order of gestation are shown in Table 1.

**Table 1:** Morbidity and mortality of preterm infants after in vitro fertilization (IVF) and after natural conception (non-IVF)

**Variable**	**IVF group n =112**	**Non-IVF group n =405**	**p-value**
*Maternal age	30 (23–39)	28 (16–46)	**<0.001**
*Gravidity	2 (1–3)	2 (1–6)	**<0.001**
**Preeclampsia	16 (14)	81 (20)	0.2
Male/female (n)	50/62	252/153	0.2
**Intrauterine growth retardation	7 (6)	51 (13)	0.1
**Cesarean section	93 (83)	326 (80)	**0.015**
**Meconium-stained amniotic fluid	1 (1)	5 (1)	0.7
**Preterm premature rupture of fetal membranes	15 (13)	63 (16)	0.8
**Outborn newborn	6 (5)	42 (10)	0.1
**No prenatal maternal management	3 (3)	19 (5)	0.1
**Antenatal steroid	73 (18)	96 (24)	**<0.001**
*Gestational age (week)	31 (23–36)	32 (22–36)	**<0.001**
*Birth weight (g)	1770 (480–4060)	1980 (380–4200)	**<0.001**
**Multiple birth			
Twins	57 (51)	56 (14)	**<0.001**
Triplets	30 (27)	10 (3)	
Quadruplets	-	1	
**Necessitating mechanical ventilation at admission	48 (43)	109 (27)	**<0.001**
**Respiratory distress syndrome	48 (43)	117 (29)	**0.005**
**Bronchopulmonary dysplasia	16 (13)	36 (9)	**<0.001**
**Intraventricular hemorrhage			**<0.001**
Grade 1	14 (13)	36 (9)	
Grade 2	7 (6)	12 (3)	
Grade 3	4 (4)	4 (1)	
Grade 4	3 (3)	3 (1)	
**Patent ductus arteriosus	19 (17)	42 (10)	
Ligation	2	1	
Ibuprofen treatment	10	14	**<0.001**
Indomethacin treatment	6	14	
**Necrotizing enterocolitis			
Grade 1	11 (10)	34 (9)	
Grade 2	7 (6)	21 (5)	**0.001**
Grade 3		2 (1)	
** Retinopathy of prematurity			
Grade 1	6 (5)	5 (1)	0.073
Grade 2	2 (2)	8 (7)	
Grade 3	2 (2)	4 (1)	
**Sepsis			
Early	28 (25)	64 (16)	0.052
Late	12 (11)	37 (9)	
*Hospitalization time	9 (0–97)	8 (0–97)	0.064
**Mortality	7 (6)	36 (9)	0.2

* Results are presented as median (minimum-maximum).

** Results are presented as number and percentage, n (%).

p < 0.05 is statistically significant. *Mann–Whitney U test or **chi-squared test was used.

In vitro fertilization cases were compared to non-IVF cases for prenatal findings preterm newborns (Table 2). In the IVF group, gestational age and birth weight were significantly lower than in the non-IVF group. Additionally, in the IVF group, multiple births, cesarean section and antenatal steroid administration were significantly higher than in the non-IVF group (p < 0.05) (Table 1). In the non-IVF group, the number of out born newborns and the lack of or poor prenatal maternal management was significantly higher than in the IVF group. Compared to the IVF group, non-life-threatening and life-threatening congenital anomalies were significantly higher in the non-IVF group. The incidence of non-life-threatening congenital heart disease in the non-IVF group was not different from the IVF group. In the preterm IVF subgroup, respiratory distress syndrome, PDA and IVH were also significantly higher than in the non-IVF preterm subgroup (p < 0.05) (Table 1). Early or late neonatal sepsis, BPD, NEC ROP, hospitalization time and mortality in preterm infants born after IVF were not different from those in the non-IVF group (Table 1). When newborns at gestational age <32 weeks were compared, only multiple birth and IVH were significantly higher in the IVF subgroup when compared to the preterm non-IVF subgroup (Table 3). In only preterm infants; using a binary logistic regression analysis, gestational age, the 5-minute Apgar score and congenital anomalies (non-life-threatening and life-threatening) were found to affect mortality. IVF has no impact on newborn mortality especially preterm mortality and morbidity (Table 2 and 4). In only preterm newborns gestational age predicted the requirement for surfactant replacement therapy, whereas IVF, gestational age, and late surfactant replacement therapy predicted the need for mechanical ventilation at admission with binary logistic regression analysis (Table 2). Only gestational age had an effect on PDA and IVH in the same logistic regression analysis (Table 2). Multiple pregnancies and PROM did not have effects on mortality, the incidence of PDA or IVH, or the need for surfactant replacement therapy and mechanical ventilation (Table 2).

**Table 2:** Risk factors for morbidity in preterm newborns

	**Surfactant administration**		**Necessitating mechanical ventilation**		**PDA**		**IVH**	
**Independent variables**	**P value**	**OR (%95CI)**	**P value**	**OR (%95CI)**	**P value**	**OR (%95CI)**	**P value**	**OR (%95CI)**
**Constant**	**<0.001**		**<0.001**		**<0.001**		**<0.001**	
IVF	0.2	1.02 (0.6-1.6)	**0.023**	2.1 (1.1-4.2)	0.12	2.02 (0.83-4.91)	0.21	1.65 (0.75-3.63)
Gestational age	**<0.001**	0.76 (0.68-0.82)	**0.005**	0.87 (0.81-0.94)	**<0.001**	0.67 (0.6-0.73)	**<0.001**	0.72 (0.65-0.79)
SGA	0.30	0.6 (1.2-1.5)	0.63	0.9 (0.4-1.92)	0.62	1.2 (0.49-3.14)	0.062	2.1 (0.97-4.8).
pPROM	0.71	087 (0.42-1.81)	0.81	1.07 (0.58-1.97)	0.31	0.66 (0.29-1.51)	0.41	0.75 (0.36-1.5)
Preeclampsia	0.1	0.76 (0.45-1.2)	0.86	0.95 (0.6-1.98)	0.23	1.6 (0.8-3.1)	0.057	0.44 (0.2-0.99)
5-minute Apgar score	0.81	1.04 (0.98-1.1)	**0.011**	0.93 (0.88-0.98)	0.35	1.03 (0.9-1.12)	0.67	1.02 (0.95-1.09)
Duration of surfactant administration			**<0.001**	1.08 (1.05-1.11)	0.31	1.01 (0.98-1.03)	0.095	1.02 (1.004-1.05)
Multiple gestation	0.33	0.7 (0.4-138)	0.4	0.82 (0.51-13)	0.45	0.67 (0.3-1.5)	0.66	1.2 (0.5-2.2)

Binary (enter) logistic regression analysis was used.

Dependent variable: surfactant administration, necessitating mechanical ventilation, PDA, IVH.

Independent variables: IVF, gestational age, SGA, pPROM, preclampcia, Apgar score at 5 minutes, duration of surfactant administration, multiple gestations.

IVF, in vitro fertilization; IVH, intraventricular hemorrhage; pPROM, preterm premature rupture of membrane; SGA, small for gestational age.

**Table 3:** Characteristics of the in vitro fertilization (IVF) and non-IVF premature newborns with gestational age <32 weeks

**Variable**	**IVF group n =40**	**Non-IVF group n =113**	**p value**
*Maternal agec	28 (20–43)	29 (20–36)	**0.001**
*Gravidity	1	1 (1–6)	**0.024**
**Preeclampsia	9 (23)	28 (25)	0.7
Male/female (n)	22/18	62/51	0.9
**Multiple birth			
Twins	20 (50)	13 (12)	**<0.001**
Triplets	10 (25)	3 (2.7)	**<0.001**
*Gestational age (weeks)	28 (22–31)	29 (23–31)	**0.034**
*Birth weight (grams)	1180 (550–2350)	1100 (380–2370)	0.2
**Intrauterine growth retardation	1 (3)	16 (14)	**0.028**
**Cesarean section	32 (80)	85 (75)	0.54
**Preterm premature rupture of fetal membranes	13 (33)	23 (20)	0.12
**Out born newborn	2 (5)	13 (12)	0.23
**No or prenatal maternal management	40 (100)	106 (94)	0.27
**Antenatal steroid	28 (70)	74 (36)	**0.002**
**Doppler	3 (8)	21 (19)	0.24
**Resuscitation	10 (25)	29 (26)	0.8
**Oligohydramnios	9 (23)	24 (21)	0.86
**Congenital anomaly, non-life-threatening/ life-threatening	1/0 (3)	6/0 (5)	0.67
**Congenital heart anomaly	4 (10)	6 (5)	0.32
*1-minute Apgar score	(0–10)	(1–9)	0.6
*5-minute Apgar score	(0–10)	(1–9)	0.9
**Respiratory distress syndrome	33 (83)	84 (79)	0.05
**Bronchopulmonary dysplasia	16 (40)	28 (25)	0.068
**ROP			
Grade 1	6 (15)	5 (4)	0.1
Grade 2	2 (5)	6 (5)	
Grade 3	2 (5)	3 (3)	
**Intraventricular hemorrhage			
Grade 1	11(28)	21 (19)	**<0.001**
Grade 2	7 (18)	7 (6)	
Grade 3	4 (10)	3 (3)	
Grade 4	3 (8)	3 (3)	

* Results are presented as median (minimum-maximum).

** Results are presented as number and percentage, n (%).

p < 0.05 is statistically significant. *Mann–Whitney U test or **chi-squared test was used.

**Table 4:** Risk factors for mortality in all preterm

	**Mortality**	
**Independent variables**	**P value**	**OR (%95CI)**
IVF	0.08	0.33 (0.09-1.17)
Gestational age	**<0.001**	0.71 (0.63-0.80)
SGA	0.4	0.6 (0.15-2.3)
pPROM	0.2	1.6 (0.6-3.9)
Preeclampsia	0.2	0.38 (0.1-1.9)
5-minute Apgar score	**0.006**	0.84 (0.76-0.93)
Duration of surfactant administration	0.2	1.01 (0.98-1.04)
Multiple gestation	0.36	1.48 (0.7-3.1)
Congenital hearth disease, non-life-threatening/ life-threatening	**<0.001**	9.2 (3.4-24.7)

When newborns at gestational age <32 weeks were compared using a binary logistic regression analysis, gestational age was the only risk factor for IVH (Table 5). Multiple gestations and antenatal steroid administration did not have effects on IVH in this subgroup of preterm newborns (Table 5).

**Table 5:** Risk factors of intraventricular hemorrhage (IVH) in premature newborns with gestational age <32 weeks

	**IVH**	
**Independent variables**	**P values**	**OR (%95CI)**
IVF	0.054	2.58 (0.98-6.82)
Gestational age	**<0.014**	0.81 (0.68-0.95)
Multiple gestations	0.12	1.65 (0.98-6.82)
Antenatal steroid	0.57	1.07 (0.83-1.37)

Binary (enter) logistic regression analysis was used.

Dependent variable: IVH.

IVF, in vitro fertilization; IVH, intraventricular hemorrhage.

## Discussion

In accordance with the literature, premature births and multiple births were common among IVF pregnancies in our study. In the multicenter study of the Turkish Neonatal Society, the frequencies of neonatal intensive care unit admissions, PDA, IVH, surfactant administration and mechanical ventilation were higher in infants born after assisted reproductive techniques, including IVF [15]. However, unlike previous studies, IVF had no effect on mortality and morbidity in our study, despite an increased risk of preterm births and multiple births [14-24]. When newborns at gestational age <32 weeks were compared, only multiple births and IVH were significantly higher in the IVF subgroup compared to the non-IVF preterm subgroup. However, only gestational age was a risk factor for IVH using the logistic regression analysis; IVF was not. Additionally, in some recently published studies, there no effects were reported on short-term mortality and morbidity in preterm infants after IVF [25-27].

In vitro fertilization had no effect on the incidence of PDA and IVH, surfactant and mechanical ventilation requirements, BPD, or ROP. Similar to infants conceived naturally, the major risk factor for morbidity in premature babies was early gestational age. Multiple pregnancies are also known to increase the morbidity in naturally conceived, premature babies. However, similar to IVF, multiple pregnancies were not associated with mortality and morbidity in this study. Congenital anomalies, particularly imprinting-type single mutations, are more common in IVF. In this study, the increased rates of congenital anomalies, particularly cardiac anomalies, in the non-IVF group were remarkable. In our region, due to the poor or absent prenatal follow-up in mothers who conceived naturally than prenatal follow-up in mothers who conceived using IVF, the increased rate of congenital anomalies in the mothers who conceived naturally may be misinterpreted. Also antenatal screening for congenital anomalies has reduced congenital anomalies through termination of pregnancy in IVF pregnancies. But in pregnancies with natural conception our countries that have not introduced the use of prenatal diagnostic techniques and access to termination of pregnancy because of congenital anomaly have seen large increments in congenital anomaly rates, unlike those countries with more restrictive policies on pregnancy termination. Therefore it is difficult to interpret the effects of IVF on the incidence of congenital anomalies because only the infants in the intensive care unit were studied. Similarly, the results of the actual incidence of premature birth or factors related to premature birth in IVF pregnancies may be misleading. The predominance of premature newborns among IVF babies in the intensive care unit was notable, in particular, extreme prematurity (birth before 29 weeks gestation) [28]. The prognosis for these very preterm infants is poor, and there is a very low chance of intact survival if born before 26 weeks [20]. The risk of cerebral palsy in twins has been estimated to be four times that of singletons, and even moderate prematurity is associated with long-term educational and behavioral problems and infant death [20,29-32].

Several studies have demonstrated that premature birth and congenital anomaly rates were increased in IVF pregnancies when compared to naturally conceived pregnancies [3-14]. However, recent publications did not report an increase in preterm births or SGA [27]. In this study by Messerschmid et al., multiple pregnancies and IVF did not have effects on preterm mortality and morbidity, as in our study [27]. They found only that the initial venous pH value was higher (7.25 vs 7.23) in multiples with weights between 1000–1499 g after IVF than naturally conceived infants. They suggested that this small difference does not appear to be relevant from a clinical standpoint [27]. We observed a higher rate of multiples after IVF than after natural conception, as in their study. Additionally, we did not assess the number of monozygotic multiples, as in their study. According to the results of 15 different national obstetrics centers, mortality is approximately 15% in twin pregnancies, at least in one of the twin pairs. The perinatal mortality ratio is 107 per thousand. Discordant females born before the 30th gestational week have the highest mortality risk [33]. Both morbidity and mortality are known to be higher in monozygotic twins compared to dizygotic twins [13]. Therefore, Messerschmid et al. concluded that they expect the outcome of multiples after IVF to be better than in multiples after natural conception [27]. However, we may suggest that the cause of the similar morbidity and mortality rates after IVF and natural conception is related to advanced therapeutic measures in newborn intensive care units because we found that multiple pregnancies were not a risk factor for increased mortality and morbidity.

There are several limitations to this study, including the small sample size of premature newborns born before 32 weeks of gestation. However, IVH was significantly higher in the IVF subgroup than the non-IVF preterm subgroup when newborns at gestational age <32 weeks were compared. It is difficult to adjust for IVH in the IVF group because multiple births are more common in the IVF group. However, when newborns at gestational age <32 weeks were compared using a regression analysis, only gestational age was found to be a risk factor for IVH. Therefore, we conclude that gestational age has an effect on IVF. Two or three eggs are fertilized in each treatment in our IVF center. Therefore, if one egg is fertilized in each treatment in our center, we may decrease the rate of premature birth and IVH.

The main drawback of our study is that the study population did not include the general population; it only included the babies in the intensive care unit. Therefore, the factors of infertility and its effects on preterm births and anomalies were not investigated. In the light of these studies and the similar data that we obtained, studies that investigate the effects of antenatal follow-up and the major factors of infertility on mortality and morbidity are required rather than studies on the use of IVF methods. Finally, these conclusions can be reached only by appropriately designed and powered randomized studies.

## Conclusion

On the basis of our study, we conclude that IVF appears to be associated with premature delivery and the known risks associated with prematurity.

## Competing interests

The authors declare that they have no competing interests.

## Authors’ contributions

GT statistically analyzed and wrote manuscript, designed study. GT, ED, AEA, AG, ASG gave your data for study. All authors read and approved the final manuscript.
